# PRESIDENTIAL SYMPOSIUM: EDUCATIONAL NEEDS AND STRATEGIES ACROSS GSA: BUILDING BRIDGES WITHIN THE SOCIETY

**DOI:** 10.1093/geroni/igad104.0936

**Published:** 2023-12-21

**Authors:** Tina Newsham, Joann Montepare

## Abstract

Across GSA member groups, we teach, train, and mentor in a variety of ways and for different purposes. Education is a tie that connects us across the Society. In this AGHE Presidential Symposium, representatives from the different GSA membership sections will discuss the educational needs of their members and share strategies they employ with their students, employees, mentees, and others to ensure the best preparation of the workforce. Researchers, care providers, educators, advocates, and others must be well-prepared to address the opportunities and demands of an aging society. Tamar Shovali, from Behavioral and Social Sciences, will present empirically supported approaches to intergenerational communication and training to foster relationships between aging individuals and their communities. Biological Sciences member LaDora Thompson will highlight current educational efforts in translating geroscience discoveries into new medical advances. Health Sciences member Matt Peterson will present strategies, challenges and opportunities learned from a currently funded health sciences mentoring program. Social Research, Policy, and Practice member Nancy Kusmaul will discuss efforts to shape policy to enhance geriatrics and gerontology education. AGHE Chair Joann Montepare will serve as discussant, exploring how AGHE can build more bridges by integrating educational efforts in several unique ways. Collectively, presenters in this session will highlight the connections and opportunities for collaboration across GSA member groups.

